# The relationship between immune fitness and saliva biomarkers of systemic inflammation

**DOI:** 10.1016/j.bbih.2023.100660

**Published:** 2023-06-30

**Authors:** Kiki EW. Mulder, Evi C. van Oostrom, Marjolijn CE. Verheul, Pauline A. Hendriksen, Suzan Thijssen, Mara AP. Diks, Aletta D. Kraneveld, Johan Garssen, Joris C. Verster

**Affiliations:** a Division of Pharmacology, Utrecht Institute for Pharmaceutical Sciences, Faculty of Science, Utrecht University, 3584CG, Utrecht, the Netherlands; b Global Centre of Excellence Immunology, Nutricia Danone Research, 3584CT, Utrecht, the Netherlands; c Centre for Human Psychopharmacology, Swinburne University, Melbourne, VIC, 3122, Australia

**Keywords:** Immune fitness, Assessment, Cytokines, c-reactive protein, Immunoglobulin A, Sex differences

## Abstract

**Purpose:**

It is vital that Immune fitness, i.e., how well the immune system functions and reacts to challenges, can be reliably be examined. The current study aimed to compare immune fitness with assessments of saliva biomarkers of systemic inflammation.

**Methods:**

N = 108 healthy young adults (18–30-year-old students of Utrecht University, the Netherlands) participated in the study. A saliva sample was collected for biomarker assessment (Interleukin (IL)-1β, IL-6, IL-8, IL-10, immunoglobulin A (IgA), and tumor necrosis factor-alpha (TNF-α), and c-reactive protein (CRP). Additionally, a survey was completed to assess immune fitness, mood, mental resilience, and quality of life. The correlations between the biomarker assessments, immune fitness and mood were determined.

**Results:**

No significant correlations between immune fitness and biomarkers of systemic inflammation were found. Significant sex differences in correlations with immune fitness were demonstrated for loneliness (significant only in men) and fatigue (significant only in women). For both sexes, immune fitness correlated significantly with anxiety, mental resilience, and quality of life.

**Conclusion:**

No significant correlations were found between immune fitness and saliva biomarkers of systemic inflammation. Immune fitness correlated significantly with anxiety, mental resilience, and quality of life. Sex differences were demonstrated in the relation of immune fitness with loneliness and fatigue. Future research should further investigate factors that may influence the relationship between immune fitness, mood, and biomarkers of systemic inflammation, including underlying psychological mechanisms of possible sex differences.

## Highlights

• The study compared immune fitness with assessments of saliva biomarkers of systemic inflammation.
• No significant correlations were found between the biomarker assessments and immune fitness.
• Immune fitness correlated significantly with mental resilience, anxiety, and quality of life.
• Sex differences were demonstrated in the relation of immune fitness with mood.

## 1 Introduction

Immune fitness can be defined as the inbuilt capacity to establish an appropriate immune response to external health challenges, thereby preventing or resolving disease (Verster et al., 2023a). A recent study estimated the annual costs of reduced immune fitness for the Dutch economy at 10.4 billion euro (Sips et al., 2023). Given this, it is vital that immune fitness, i.e., how well the immune system functions, can be assessed reliably and accurately. Currently, there are two ways to evaluate immune fitness, both subjective and objective, and each of these approaches has advantages and disadvantages.

Subjective assessment of immune fitness can comprise a single-item assessment. For example, asking a person to rate his/her immune fitness on an 11-point scale ranging from 0 (very poor) to 10 (excellent) (Verster et al., 2023a; Van Schrojenstein Lantman et al., 2017). Single-item assessments for real-time or retrospective assessments are ideal for situations in which time constraints are typical, such as clinical practice, surveys, or clinical trials, to monitor the effects of interventions. In addition, single-item assessments can be a solution in case of space constraints (e.g., screening checklists or mobile apps). An important advantage of single-item assessment is that it automatically encompasses the total concept of immune fitness, including its impact on the individual's well-being, mood, and quality of life (Wilod Versprille et al., 2019; Guidance for Industry, 2009; García-Gutiérrez et al., 2020; Verster et al., 2021a). Several studies have successfully applied the single-item assessment of immune fitness and revealed significant correlations with health and mood outcomes (Baars et al., 2019; Verster et al., 2021b). However, a disadvantage of single-item assessments is that they provide no insight into possible underlying causes of the level of immune fitness. For that purpose, multiple item scales may be more informative. For example, the Immune Status Questionnaire (ISQ) retrospectively assesses past year's frequency of occurrence of immune-related complaints such as headache and common cold (Wilod Versprille et al., 2019). Unfortunately, given the answering possibilities of the ISQ, this scale is not suitable for momentary assessments, nor does it assess severity (Verster et al., 2023a).

An objective way to evaluate immune fitness is to assess biomarkers of the immune system, such as c-reactive protein (CRP), cytokines, or antibodies. Traditionally, these assessments are performed in blood samples by counting the type and number of immune cells or mediators. The advantage of analyzing biomarkers is that it yields objective outcomes that can be compared to reference (normal) values from historical data. A biomarker profile can then be compared to pre-set cut-off values of health and disease to determine the clinical relevance of the observations. Hence, it is an objective way to monitor disease progress or the efficacy of an intervention in clinical trials. In addition, only for a small number of biomarkers (e.g., blood glucose or bodyweight), the outcome of the biomarker assessment is readily available. However, a disadvantage of many other biomarker assessments is that they may be expensive, are time-consuming, and sample collection is often invasive (Mayeux, 2004). In addition, invasive assessments may be a burden to many patients or even be feared, as these assessments can be painful (McLenon and Rogers, 2019). As an alternative, biomarker assessments can be conducted by applying non-invasive techniques, such as collecting saliva, stool, or urine samples instead of blood. Although these types of assessments are non-invasive, the data collection of these alternative samples still requires considerable time and effort (Celec et al., 2016).

Direct comparisons between immune fitness and biomarkers of systemic inflammation are scarce and therefore need more attention and research. Petrie et al. (1999) found that perceptions of immune functioning were unrelated to the concentrations of serum antibodies or blood lymphocytes (serum immunoglobulin A (IgA), IgG, and IgM antibodies, and cluster of differentiation 3 (CD3), CD4, CD8, and CD16 lymphocytes). Instead, they found that not these biomarkers of the immune system but feelings of vigor and fatigue were the main determinants of individuals’ perception of their immune fitness. The authors explained their findings by the notion that tired and run-down individuals are more susceptible to infections and illness.

To further investigate this, the purpose of the current study was to compare immune fitness with mood and saliva biomarker assessments of pro-and anti-inflammatory cytokines (inflammation) and C-reactive protein (CRP) (systemic inflammation).

## 2 Methods

The study was approved by the Science-Geo Ethics Review Board of Utrecht University (protocol ID: S-21525, date of approval: Nov 21, 2021), and all participants provided written informed consent. The study was conducted in December 2021. N = 108 healthy young adults (18–30-year-old students of Utrecht University, the Netherlands), of which 31 men and 77 women, participated in the study. Participants we reimbursed 20 euros for their participation. A saliva sample was collected for biomarker assessment, and a survey was completed to assess demographics (age and sex), immune fitness, mood, mental resilience, and quality of life. The saliva sample was taken between 9 a.m. and 6 p.m. The participants were requested not to eat within 30 min before taking the test. As the outcomes of biomarker assessments and subjective assessments of mood and health may vary throughout the day (Scheiermann et al., 2013), both assessments were conducted directly after each other.

### 2.1 Subjective assessments

Mood was assessed via 1-item scales including “stress”, “anxiety”, “depression”, “fatigue”, “loneliness”, “hostility”, “being active”, “optimism”, and “happiness”. All items were scored on a scale ranging from 0 (absent) to 10 (extreme) (Verster et al., 2021a, 2023b). In a similar way, quality of life, mental resilience (i.e., the ability to bounce back), and immune fitness were assessed with single-item scales ranging from 0 (very poor) to 10 (excellent) (Verster et al., 2021a, 2023b).

#### 2.1.1 Biomarkers of the immune system

A saliva sample was collected by the passive drool method, using SalivaBio's Saliva Collection Aid (Salimetrics, State College, PA, USA). The timing of saliva sample collection varied throughout the day, depending on the arrival time of the participant. Eating or drinking was not allowed within 30 min before saliva collection. Saliva samples were stored at −80 Celsius using EDTA collection tubes (Greiner Bio One, Kremsmünster, Austria), including a protease inhibitor cocktail (Merck, Darmstadt, Germany). Immunoglobulin A (IgA) was assessed by enzyme-linked immunosorbent assay (Elisa), following standard operating procedures (Dingess et al., 2021). For CRP, interleukin (IL)-1β, IL-6, IL-8, IL-10 and tumor necrosis factor-alpha (TNF-α), the saliva concentrations (in pg/ml) were assessed by multiplex immunoassay (customized ProcartaPlex Immunoassay, ThermoFisher Scientific, Waltham, USA), using standard procedures described elsewhere (Van de Loo et al., 2021). The measurement of the samples was performed as a single measurement, while the standards were measured in duplicate. To evaluate differences between assay performance, the percent coefficient of variation (CV%) value was used. CV% is defined as (standard deviation/mean) × 100 (expressed as percentage). In general, a CV of less than 10% is considered acceptable (Luminex. xMAP Cookbook, 2023; Lee et al., 2006). For the standards 1 to 6, a CV% between 1.6 and 9.3 was observed. Standard 7 showed a CV% of 30.2 and, therefore, standard point 6 was used as limit of detection (LOD) of this Luminex assay. For assessments below the LOD, half the LOD value of the assay was included in the dataset. If more than 30% of the cytokine assessments were below the LOD value, the cytokine was excluded from the statistical analysis.

#### 2.1.2 Statistical analysis

Statistical analyses were conducted with SPSS (IBM Corp. Released, 2013. IBM SPSS Statistics for Windows, Version 28.0. Armonk, NY, USA: IBM Corp.). Mean and standard deviation (SD) were computed for all variables. Differences between men and women were evaluated with the Independent-Samples Mann-Whitney *U* Test, and considered significant if p < 0.05 (two-sided). Spearman's correlations were computed between immune fitness, the biomarkers, and the mood and quality of life assessments. This was done for the sample as a whole and separate for the subsamples of men and women. Correlations were considered significant if p < 0.0011, applying a Bonferroni's correction for multiple comparisons. Possible sex differences between the correlations were investigated with the Fisher r-to-z transformation (online calculator, available at http://vassarstats.net/rdiff.html). Sex differences were considered significant if p < 0.0011, applying a Bonferroni's correction for multiple comparisons (two-tailed).

## 3 Results

N = 108 young adults participated in the study. The saliva sample analysis revealed that for IL-6, IL-10 and TNF-α, more than 30% of participants had values that were below the limit of detection. Thus, these cytokines could not be reliably detected in saliva and were not further considered. In addition, the saliva of one participant was not suitable for processing (due to the thickness of the sample) and was excluded from the final dataset. Table 1 gives an overview of the study outcomes. No significant sex differences were found.

**Table 1:** Summary of the study outcomes.

	Overall (N = 108)	Men (N = 31)	Women (N = 77)	p-value
Immune fitness	7.6 (1.2)	7.8 (1.3)	7.5 (1.2)	0.338
CRP (pg/ml)	155.9 (193.7)	142.9 (172.7)	161.2 (202.5)	0.631
IL-1β (pg/ml)	174.3 (212.0)	183.2 (243.4)	170.7 (199.5)	0.940
IL-8 (pg/ml)	232.1 (166.8)	250.7 (174.0)	224.5 (164.3)	0.410
IgA (pg/ml)	61445.7 (31498.0)	62438.2 (30357.9)	61046.1 (32131.9)	0.622
Stress	4.0 (2.7)	3.3 (2.7)	4.2 (2.7)	0.133
Anxiety	1.8 (2.2)	1.8 (2.0)	1.9 (2.3)	0.846
Depression	1.6 (2.0)	2.0 (2.4)	1.4 (1.9)	0.240
Fatigue	4.6 (2.4)	4.2 (2.4)	4.8 (2.3)	0.304
Loneliness	1.7 (2.2)	1.7 (2.2)	1.7 (2.2)	0.955
Hostile	0.6 (1.4)	1.1 (2.0)	0.4 (1.0)	0.101
Optimism	6.3 (1.5)	6.2 (1.4)	6.4 (1.5)	0.430
Happiness	6.6 (1.5)	6.3 (1.9)	6.8 (1.2)	0.303
Being active	6.2 (1.6)	6.2 (1.5)	6.2 (1.6)	0.659
Mental resilience	6.8 (1.2)	6.9 (1.4)	6.8 (1.1)	0.649
Quality of life	7.5 (1.0)	7.6 (1.0)	7.5 (1.0)	0.809

None of the differences between men and women was statistically significant. Abbreviations: CRP = c-reactive protein, IL = interleukin, IgA = Immunoglobulin A.

Correlations with immune fitness and their significance are summarized in Table 2. No significant correlations were found between immune fitness and the biomarkers. For mood items, anxiety and fatigue correlated significantly with immune fitness. Also, mental resilience, being active, and quality of life correlated significantly with immune fitness. Several sex differences were observed. In men, loneliness correlated most robust with immune fitness, whereas this correlation was not significant in women). The strongest correlation for women was found between immune fitness and fatigue, whereas this correlation was not significant in men. Finally, for both sexes, mental resilience and quality of life significantly correlated with immune fitness. No significant correlations were found between the biomarker assessments and mood, mental resilience, or quality of life.

**Table 2:** Correlations with immune fitness.

	Overall		Men		Women	
	r	p-value	r	p-value	r	p-value
CRP	−0.157	0.106	0.092	0.624	−0.235	0.041
IL-1β	−0.036	0.713	0.200	0.281	−0.131	0.258
IL-8	0.010	0.915	0.186	0.315	−0.060	0.607
IgA	0.086	0.373	0.178	0.337	0.044	0.703
Stress	−0.216	0.025	−0.195	0.292	−0.217	0.058
Anxiety	−0.314	<0.001 *	−0.258	0.162	−0.337	0.003
Depression	−0.205	0.033	−0.173	0.353	−0.241	0.035
Fatigue	−0.327	<0.001 *	−0.262	0.154	−0.356	0.001 *
Loneliness	−0.250	0.009	−0.585	<0.001 *	−0.109	0.344
Hostility	−0.275	0.007	−0.417	0.030	−0.226	0.061
Optimism	0.169	0.081	0.129	0.490	0.193	0.092
Happiness	0.143	0.140	0.093	0.620	0.172	0.134
Being active	0.262	0.006	0.151	0.418	0.323	0.004
Mental resilience	0.399	<0.001 *	0.456	0.010	0.378	<0.001 *
Quality of life	0.444	<0.001 *	0.616	<0.001 *	0.376	<0.001 *

Spearman's correlations are presented. Significant correlations (*p* < 0.0011, two-tailed, after Bonferroni's correction for multiple comparisons) are indicated by *. No significant sex differences were observed. Abbreviations: CRP = c-reactive protein, IL = interleukin, IgA = Immunoglobulin A.

The observed biomarker concentrations are in line with those that have been published previously (Van de Loo et al., 2021; Plank et al., 2021). However, a possible impact on the time of day of taking the assessments has been suggested (Scheiermann et al., 2013). To evaluate whether a time of day effect was present in the current study, the immune fitness scores and biomarker concentrations of individuals that were tested in the morning (between 09:00 and 12:00 h), midday (between 12:00 and 15:00 h), and the afternoon (between 15:00 and 18:10) were compared. The analysis revealed no significant differences between the time periods for immune fitness, CRP, and IL-1β. Whereas the IL-8 concentrations in the morning (mean ± SD = 174.1 ± 125.9 pg/ml) and afternoon (mean ± SD = 236.9 ± 185.9 pg/ml) did not statistically differ from each other, the concentration of the morning assessments was significantly lower than the midday assessment (mean ± SD = 269.5 ± 167.7 pg/ml, p = 0.019). The midday and afternoon assessments of IL-8 did not significantly differ from each other.

Finally, a regression analysis was conducted to determine to what extend the combination of assessed biomarkers predict immune fitness. The analysis yielded a poor model predicting immune fitness for only 3% (R^2^), with CRP as the only indicator variable.

## 4 Discussion

This study compared immune fitness with selected saliva biomarkers of systemic inflammation. In addition, associations with mood, mental resilience, and quality of life were assessed. No significant sex differences were found for immune fitness and biomarkers of systemic inflammation. In addition, after Bonferroni's correction for multiple comparisons, the correlations between immune fitness and biomarkers were not statistically significant. Significant correlations were found between immune fitness and mood, mental resilience and quality of life, but these variables did not correlate significantly with biomarker assessments.

Sex-specific associations were found between immune fitness and mood. In men, loneliness correlated most robust with immune fitness, whereas this correlation was not significant in women. The strongest correlations for women was found between immune fitness and fatigue, but this correlation was not significant in men.

When interpreting the data, it is important to take into account that due to the immune system's complexity, any correlation between immune fitness with a single biomarker of the immune system biomarkers will be modest at best (Verster et al., 2023a). Indeed, the immune system comprises the complex interplay between a large number of cells and mediators that together determine one's immune fitness. Therefore, it is not likely that a single cytokine or other biomarkers of immune functioning can adequately represent the whole concept of (perceived) immune fitness (Verster et al., 2023a). However, the modest but nonsignificant correlations observed for CRP in the current study, supports the notion that differences in the concentration of a single biomarker do provide a directional indication of one's immune fitness. Therefore, the assessment of both biomarkers and immune fitness has an additive value that contributes to the understanding of a person's immune fitness. This hypothesis is in line with previous studies that found only modest associations between biomarker concentrations and subjective assessments of general health (Lekander et al., 2004; Nakata et al., 2010; Christian et al., 2011; Leshem-Rubinow et al., 2015; Kananen et al., 2011).

Similar to immune fitness, general health is a broad and complex concept that is unlikely to be captured adequately by a single biomarker. Indeed, previous research revealed significant correlations between self−rated health and immune biomarkers in both patients (Lee et al., 2006) and healthy volunteers (Nakata et al., 2010; Christian et al., 2011; Leshem-Rubinow et al., 2015). A study by Kananen et al. (2011) reviewed data on the association of self-rated health with 150 biomarkers and found significant correlations for 57 biomarkers. Most notably, self-rated health associations were found with biomarkers such as CRP (for inflammation), cholesterol, and HbA1c (for lipid and glucose metabolisms). However, the observed correlations were modest, illustrating that these biomarkers do not fully encompass the whole concept of ‘health’. The latter is also evident from research into other health concepts that can be considered too complex to be captured by a single biomarker, such as the alcohol hangover (Van de Loo et al., 2021), risk-taking behavior (Van den Bos et al., 2014), and immune fitness (Verster et al., 2023a). Taken together, it is understandable that the observed correlations between immune fitness and biomarkers of systemic inflammation are modest at best and did not reach statistical significance in the current study. In the current study, regression analysis revealed that combining the different biomarkers into one model did not relevantly predict immune fitness.

The fact that only young and healthy subjects participated in the study reduces the variance in assessment outcomes of immune fitness and biomarkers, and this may be another reason why no significant correlations were observed. These findings are not in line with previous research. It is known that males and females differ in innate and adaptive immune responses and their immunological responses to antigens (Klein and Flanagan, 2016). Importantly, these immunological sex differences affect the susceptibility of individuals to infections, how they react to immunization, and how frequently they develop autoimmune disorders and cancers (Lekander et al., 2004; Klein and Flanagan, 2016)^..^ In line, women and men show significant differences in ratings of immune fitness. Previous studies in much larger samples consistently found that women score significantly poorer on the single-item immune fitness scale than men. For example, Van Schrojenstein Lantman et al. (Van Schrojenstein Lantman et al., 2017) examined immune fitness among 2489 young adults, 18–30 years old (N = 414 men and N = 2075 women). The analysis revealed a significantly better (p < 0.001) mean ± SD immune fitness in men (8.0 ± 1.3) compared to women (7.4 ± 1.4). Kiani et al. (Van den Bos et al., 2014) examined immune fitness among the general population, 18–96 years old (N = 370 men and N = 650 women). The analysis also revealed a significantly better (p < 0.001) mean ± SD immune fitness in men (7.6 ± 1.8) compared to women (7.0 ± 2.1). Given these findings, it was expected that women also reported a poorer immune fitness in the current study than men. Indeed, the mean ± SD score of women (7.5 ± 1.3) was lower than that of men (7.8 ± 1.2). Important to note is that the sex difference did not reach statistical significance due to the relatively small sample size. The same studies (Van Schrojenstein Lantman et al., 2017; Kiani et al., 2021) also found that women score significantly poorer than men on several mood items. This is in line with the observations in the current study.

The current study has some limitations that should be mentioned. First, the cross-sectional design of this research and correlational analyses do not allow the identification of casual relationships. Second, the sample comprised young, relatively healthy students. Therefore, the study should be replicated in non-students, other age groups, and patient populations. Third, only a limited number of biomarkers of systemic inflammation were assessed. Future studies should measure a greater variety of biomarkers. Fourth, the timing of saliva sample collection varied throughout the day. This may, to some extent, have influenced the biomarker concentrations (as shown in the case of IL-8). Future studies should therefore collect biomarker samples at a fixed time of day. Fifth, given that several biomarkers could not be (reliably) detected in saliva, preferably, the biomarker assessments should also be conducted in blood. This will allow a direct comparison with saliva biomarker assessments. Finally, the underlying mechanisms and causes of sex differences in immune fitness were in not assessed in this study. Future studies should investigate potential sex differences, including the general perception of what comprises health and disease. A recent study revealed that reduced immune fitness is associated with significant costs for the Dutch economy (Sips et al., 2023). Another study demonstrated that for future pandemic preparedness, maintaining an adequate immune fitness was the most important factor considered (Kiani et al., 2022). These findings illustrate the need for more research into the causes, consequences, prevention, and assessment of (reduced) immune fitness.

## 5 Conclusion

This study revealed no significant correlations between immune fitness and biomarkers of systemic inflammation. However, significant correlations were found between immune fitness and mental resilience, quality of life, and anxiety, and sex-specific correlations of immune fitness with fatigue (significant in women) and loneliness (significant in men).

## Ethics approval

This study was performed in line with the principles of the Declaration of Helsinki. Approval was granted by the Science-Geo Ethics Review Board of Utrecht University (protocol ID: S-21525, date of approval: Nov 21, 2021).

## Funding

The authors declare that no funds, grants, or other support were received during the preparation of this manuscript.

## Authors' contributions

All authors contributed to the study conception and design. Material preparation, data collection and analysis were performed by Kiki Mulder, Marjolijn Verheul, Evi van Oostrom, Pauline Hendriksen, Suzan Thijssen, Mara Diks and Joris Verster. The first draft of the manuscript was written by Kiki Mulder and all authors commented on previous versions of the manuscript. All authors read and approved the final manuscript.

## Availability of data and material

The datasets generated during and/or analyzed during the current study are available from the corresponding author on reasonable request.

## Consent to participate

Informed consent was obtained from all individual participants included in the study.

## Consent for publication

Not applicable.

## Declaration of competing interest

The authors declare that they have no known competing financial interests or personal relationships that could have appeared to influence the work reported in this paper. Over the past 3 years, Joris Verster has acted as a consultant/advisor for Eisai, KNMP, Red Bull, Sen-Jam Pharmaceutical, and Toast!. Aletta Kraneveld has held research grants from H2020, Nutricia-Danone, Netherlands Center of Translational Research, Lung fund, SGF/Health Holland, and NWO. Johan Garssen is a part-time employee of Nutricia Research and received research grants from Nutricia research foundation, Top Institute Pharma, Top Institute Food and Nutrition, GSK, STW, NWO, Friesland Campina, CCC, Raak-Pro, and EU. The other authors have no potential conflicts of interest to disclose.

## Data Availability

Data will be made available on request.
